# The Implantation of Sterilized Roots of Animals’ Teeth, Carrying Artificial Crowns*Abstract of a paper road before the Dental Section, American Medical Association, Philadelphia, June, 1897.

**Published:** 1897-08

**Authors:** Wm. Ernest Walker


### The Implantation of Sterilized Roots of Animals' Teeth, Carrying Artificial Crowns.*

BY WM. ERNEST WALKER, D. D. S.

Professor of Prosthetic Dentistry, Dental Department, Southern Medical College, Atlanta, Ga.

It is a deplorable fact that notwithstanding the great advance made in the treatment of the various diseases of the oral cavity, occasionally a tooth is lost in spite of all the best endeavors of the stomatologist to prevent such a calamity. It is therefore very desirable that we should be able to replace such lost organs, and often by other means, than plate or bridge-work. I need therefore make no apology for bringing before you the subject of planting teeth.

Since the introduction of the operation of implantation by Dr. Wm. J. Younger (1885), various substitutes for carrying artificial crowns—as lead capsules, porcelain teeth, double staples of metal reversed, etc.—the literature on the subject does not indicate any marked degree of success with any of the roots save natural ones.

As it is often a difficult matter to obtain a healthy root of a human tooth suitable for this purpose, it has occurred to me that we might utilize the roots of the teeth of beasts which are easily obtained from the slaughter pen, and which are, as a rule, free from disease, requiring, however, thorough sterilization. Before implanting they can be surmounted with Logan or other crowns, a cast made of the mouth and a bandage made on it by Dr. Jack's method, which I have found the most satisfactory. While I have not found any beast's teeth with roots very closely corresponding to those of human molars, this is immaterial as the socket from which a molar has been extracted requires remodeling, even to receive a human molar, for the roots of no two human molars are alike, and less cutting is necessary in using the large, single root of a bovine central incisor, all that is demanded, being

* Abstract of a paper read before the Dental Section, American Medical Association, Philadelphia, June, 1897.

the removal of the septum dividing the aloveoli of the socket. I have examined a number of heads and find that while bovine centrals are very well developed and admirably adapted to this purpose, it is not so with the lateral incisors at the age at which beef is usually slaughtered in this section of the country, the root of the lateral not being fully developed and the foramen very large.

This is not, strictly speaking, *implantation*, but a practical *transplantation* which might be practiced by some who are hesitating to drill sockets in the molar region for implantation, or who might be deterred by the difficulty of inserting a multi-rooted tooth.

I will not lengthen my paper by going into the details of the removal of pulp, sterilization, canal filling, adjusting artificial crowns, etc., for any necessary variation in the procedure from which has been published on implantation and transplantation, will naturally suggest itself to any one who will take the trouble to review the literature of the subject before undertaking the operation.

